# Biochemical reconstitution of TET1–TDG–BER-dependent active DNA demethylation reveals a highly coordinated mechanism

**DOI:** 10.1038/ncomms10806

**Published:** 2016-03-02

**Authors:** Alain R. Weber, Claudia Krawczyk, Adam B. Robertson, Anna Kuśnierczyk, Cathrine B. Vågbø, David Schuermann, Arne Klungland, Primo Schär

**Affiliations:** 1 Department of Biomedicine, University of Basel, Mattenstrasse 28, Basel CH-4058, Switzerland; 2 Department of Molecular Microbiology, Oslo University Hospital, Rikshospitalet, NO-0372 Oslo, Norway; 3 Proteomics and Metabolomics Core Facility, PROMEC, Department of Cancer Research and Molecular Medicine, Norwegian University of Science and Technology, NO-7489 Trondheim, Norway

## Abstract

Cytosine methylation in CpG dinucleotides is an epigenetic DNA modification dynamically established and maintained by DNA methyltransferases and demethylases. Molecular mechanisms of active DNA demethylation began to surface only recently with the discovery of the 5-methylcytosine (5mC)-directed hydroxylase and base excision activities of ten–eleven translocation (TET) proteins and thymine DNA glycosylase (TDG). This implicated a pathway operating through oxidation of 5mC by TET proteins, which generates substrates for TDG-dependent base excision repair (BER) that then replaces 5mC with C. Yet, direct evidence for a productive coupling of TET with BER has never been presented. Here we show that TET1 and TDG physically interact to oxidize and excise 5mC, and proof by biochemical reconstitution that the TET–TDG–BER system is capable of productive DNA demethylation. We show that the mechanism assures a sequential demethylation of symmetrically methylated CpGs, thereby avoiding DNA double-strand break formation but contributing to the mutability of methylated CpGs.

## Abstract (web summary)

Cytosine methylation is a dynamic DNA modification with the involvement of the base excision repair pathway suspected to be involved in demethylation. Here the authors show that TET1 and TDG interact to target modified bases and coordinate BER to avoid double strand breaks.

## Graphical abstract

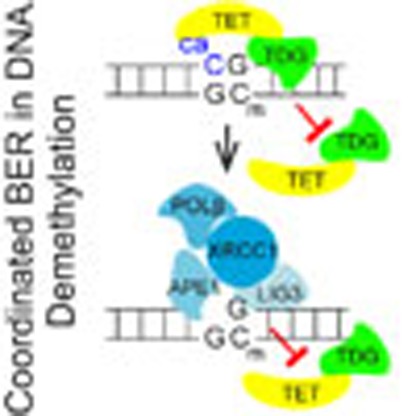

DNA methylation in mammals occurs at the C5 position of cytosines (5-methylcytosine, 5mC) and is found predominantly within CpG dinucleotides, affecting 60–90% of such sites^1^. Modulating chromatin states and thereby transcriptional activity and genome stability, DNA methylation plays an important epigenetic role in various biological processes^2^. It is generally viewed as a static DNA modification but recent research has shown that under specific circumstances, DNA methylation can be subject to dynamic change. This is best illustrated by its genome-wide erasure during early embryonic development^3^,^4^,^5^ or in maturing primordial germ cells^6^. Locus-directed DNA demethylation has also been observed in somatic cells upon triggering transcriptional activation in various ways^7^,^8^,^9^. Both passive and active pathways of DNA demethylation were proposed to operate in these contexts but the mechanisms underlying active demethylation, in particular, have remained controversial^10^.

Recent evidence substantiates an involvement of the ten eleven–translocation (TET) family of dioxygenases^11^. TET proteins oxidize 5mC to 5-hydroxymethylcytosine (5hmC), 5-formylcytosine (5fC) and 5-carboxylcytosine (5caC), all of which have been implicated as intermediates of DNA demethylation^12^,^13^,^14^,^15^. Regarding active mechanisms, 5fC and 5caC appear of particular relevance, as these bases are substrates for the thymine DNA glycosylase (TDG), a DNA repair protein with an ability to excise various cytosine and 5mC base derivatives from DNA^12^,^16^,^17^,^18^. Thus, TET and TDG together constitute catalytic activities capable of oxidation and removal of 5mC in DNA. This biochemical reasoning is supported by the phenotype of TET^19^,^20^,^21^,^22^ and TDG knockout mice^23^,^24^,^25^, as well as embryonic stem cells, all showing aberrations in DNA methylation^23^,^26^,^27^,^28^,^29^.

TET and TDG thus initiate active DNA demethylation by oxidation and excision of 5mC in DNA and the anticipated downstream events will be the excision and repair of the resulting abasic site (AP-site) by the DNA base excision repair (BER) system. An engagement of the core BER system implies that the AP-site is first incised by an AP endonuclease (that is, APE1), which generates a DNA single-strand break that then engages, through activation of poly (ADP-ribose) polymerase 1, the X-ray repair cross-complementing protein 1 (XRCC1), DNA ligase 3 (LIG3) and DNA polymerase β (POLβ) for DNA gap filling with an unmethylated C and ligation^30^. Although this mechanism is plausible and widely accepted, there is little evidence supporting a direct link between TET and BER; a productive action of TET with the BER system on a 5mC substrate has not been shown, nor have the basic mechanistic features of such a process been addressed.

The aim of this study was therefore to reconstitute the full DNA demethylation system *in vitro* and to address specific properties of the DNA transactions involved. We investigated physical and functional interactions between TET1 and TDG, and tested the hypothesis that methylated DNA substrates can be converted to unmethylated DNA through oxidation and BER of 5mC. We addressed the strand specificity of the reaction, whether symmetrically modified CpGs can be demethylated without DNA fragmentation and how complex lesions such as occurring by simultaneous oxidation and deamination of opposite 5mCs within a CpG dinucleotide do affect the demethylation outcome. The data proof full functionality of a TET1–TDG–BER-based DNA demethylation system on hemi- and fully methylated DNA, and show that the molecular transactions involved are coordinated in a manner avoiding DNA fragmentation but creating a risk for mutation if deamination and demethylation events coincide within a CpG.

## Results

### TET1 and TDG interact physically

**Figure 1:**
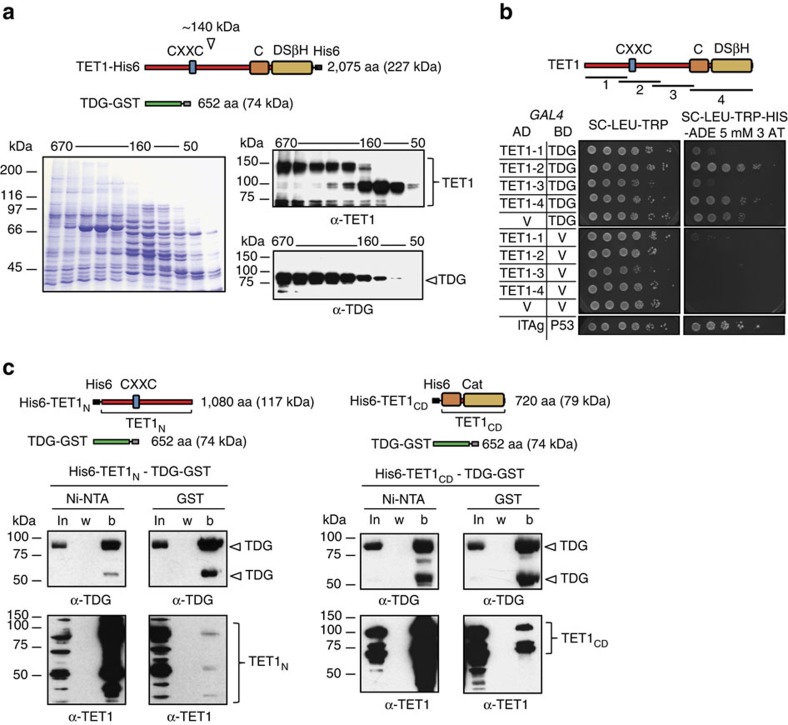
TET1 physically interacts with TDG. (**a**) Size fractionation by gel filtration at high ionic strength (500 mM NaCl) of Ni-NTA-enriched lysates of *E. coli* cells co-expressing TET1–His6 and TDG–GST from constructs indicated. Fractions were analysed by SDS–PAGE (left panel), and TET1 and TDG detected by immunoblotting (right panel); molecular weights of gel filtration standards are indicated. (**b**) Yeast two-hybrid analysis of the TET1–TDG interaction. TET1 domains cloned into the GAL4 activation domain (AD), TET1-1 (aa 1–491), TET1-2 (aa 397–931), TET1-3 (aa 870–1403) and TET1-4 (aa 1,367–2,057) are indicated at the top. Shown is the growth of serial dilutions of strains co-expressing TET1 domains fused to the AD and TDG fused to the GAL4-binding domain (BD) and respective negative controls (TET1 domains or TDG co-expressed with the vector control (V)) on permissive and selective media. The large T antigen (lTAg) and p53 fused to the AD and BD, respectively, served as a positive control. (**c**) Immunoblotting of fractions obtained from Ni-NTA and GST purifications using *E. coli* extracts co-expressing His6–TET1_N_ and TDG–GST (left panel), or co-expressing His6–TET1_CD_ and TDG–GST (right panel). Expression constructs used are indicated; TET1_N_ (aa 301–1366) and TET1_CD_ (aa 1367–2057); b, bound fraction, in, input; w, wash.

The model of TET–TDG-mediated oxidative DNA demethylation postulates a coupled action of both enzymes to facilitate an efficient removal of 5mC. To address the mode of cooperation between TET and TDG, we investigated their physical interaction, first by co-expression and affinity purification of a full-length carboxy-terminally 6His-tagged TET1 (TET1–His6) with a C-terminally glutathione *S*-transferase (GST)-tagged TDG (TDG–GST). Although co-expression with TDG positively affected full-length TET1 expression, enrichment of TET1–His6 via Ni-NTA chromatography yielded little full-length protein but prominent, presumably C-terminal fragments of ∼140–150, 90 and 60–70 kDa, possibly reflecting proteolysis of poorly structured domains. Size fractionation by gel filtration then showed that two of these fragments (140–150 and 60–70 kDa) co-eluted with full-length TDG–GST in high-molecular-weight fractions (200–600 kDa) at high ionic strength (500 mM NaCl) and down to concentrations in the 100 nM range, as assessed semi-quantitatively on the basis of immunoblot signals (Fig. 1a). This indicated the formation of stable TET1–TDG complexes. The 90-kDa TET1–His6 fragment appeared in lower-molecular-weight fractions (90–200 kDa) and only partially co-eluted with TDG–GST, indicating a weaker interaction with TDG.

To further characterize the TET1–TDG interaction, we used the yeast two-hybrid system. Four protein fragments spanning the entire TET1 polypeptide (Fig. 1b) were fused to the Gal4-binding domain (bait) and co-expressed separately with TDG fused to the Gal4 activation domain (prey) in yeast. Growth on media selecting for two-hybrid reporter gene activation indicated physical interactions between TDG and TET1 fragments 2 and 4. These results thus indicated that TET1 harbours specific TDG interaction domains in its amino terminus (amino acids (aa) 397–931) comprising the CXXC motif and in its C-terminal catalytic domain (aa 1367–2057) (Fig. 1b). We next performed co-precipitations from lysates of *Escherichia coli* cells co-expressing TDG–GST with either a His6-labelled TET1 N-terminal fragment (TET1_N_; aa 301–1366) or the TET1 catalytic domain (TET1_CD_; aa 1367–2057) (Fig. 1c). TDG–GST co-eluted from the Ni-NTA resin in the bound fraction with both TET1 fragments (Fig. 1c, Ni-NTA). The outcome was the same when we enriched for TDG–GST; both TET1 fragments co-eluted in the bound fraction after GST affinity purification (Fig. 1c, GST). The results of all protein interaction assays led us to conclude that TET1 and TDG physically interact through specific N-terminal and C-terminal TET1 domains.

### TET1_CD_ and TDG act in concert to release 5mC

**Figure 2:**
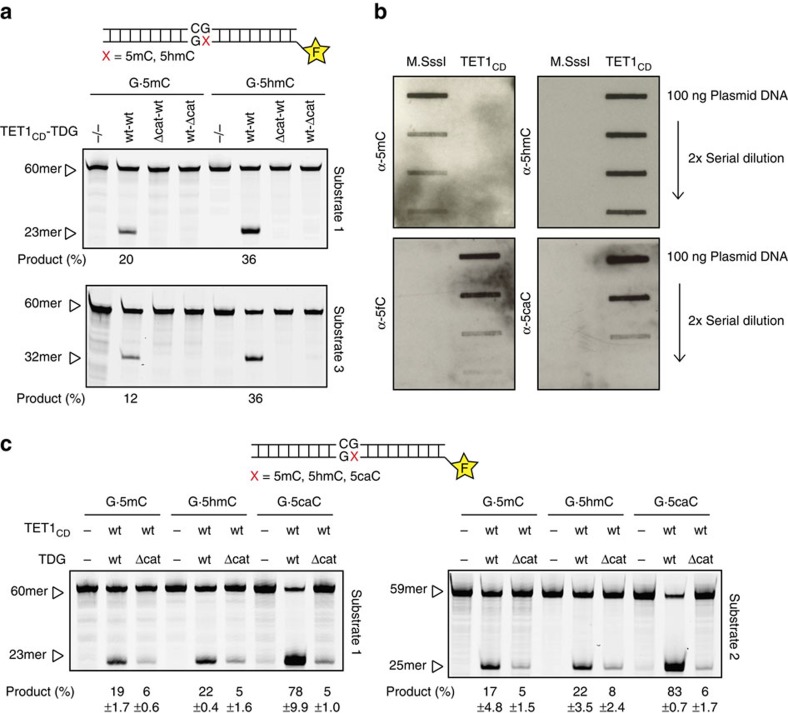
Combined TET1 and TDG activity releases 5mC through oxidized intermediates. (**a**) Base-excision activity of Ni-NTA-enriched His6–TET1_CD_–TDG–GST on synthetic DNA substrates as indicated. The ability to generate alkaline-sensitive AP-sites in substrates containing either single G·5mC or G·5hmC base pairs was assayed with enriched His6–TET1_CD_–TDG–GST consisting of wild-type proteins or respective mutant variants (His6–TET1_CD_Δcat–TDG–GST, His6–TET1_CD_–TDGΔcat–GST). Products were separated by denaturing gel electrophoresis, visualized with fluorescent scanning and quantified; positions of the 60mer substrate DNA and product fragment are indicated. (**b**) Slot blot analysis of plasmid oxidation by purified His6–TET1_CD_. *In-vitro*-methylated pUC19 plasmid DNA (800 nM) was treated with His6–TET1_CD_ (125 nM) and cytosine modifications were detected by immunblotting with specific antibodies against 5mC, 5hmC, 5fC and 5caC. (**c**) Reconstitution of 5mC/5hmC base release with purified His6–TET1_CD_ and His6–TDG proteins. DNA substrates (25 nM) containing either G·5mC, G·5hmC or G·5caC base pairs were reacted with preassembled His6–TET1_CD_–His6–TDG (50 nM), reaction products separated by denaturing gel electrophoresis, visualized and quantified. Positions of the 60mer substrate DNA and product fragments are indicated. Shown are mean values with s.d. (*n*=3).

To examine the activity of the TET–TDG complex, we co-expressed the catalytic domain of TET1 (His6–TET1_CD_) and TDG–GST, as well as combinations of their catalytic-dead variants ((His6–TET1_CD_Δcat (H1652Y; D1654A) with TDG–GST; His6–TET1_CD_ with TDGΔcat–GST (N151A)) in *E. coli* and enriched the complexes by Ni-NTA chromatography (Supplementary Fig. 1). We then measured catalytic activities in a base release assay^31^ with two fluorescein-labelled synthetic 60-bp DNA substrates containing a single 5mC or 5hmC. Incubation of the enriched His6–TET1_CD_–TDG–GST fraction with both DNA substrates (1 h, 37 °C) generated a substantial amount of DNA incisions at the position of the modified cytosines (Fig. 2a). This 5mC/5hmC excision activity was not detectable when either of the two proteins was mutated at its catalytic site, establishing that the excision of 5mC and 5hmC from DNA requires the catalytic activities of both TET1 and TDG.

To confirm that the intermediates generated in these assays were oxidized 5mC or 5hmC, we examined the DNA products generated by His6–TET1_CD_ in the absence of TDG. Purification of His6–TET1_CD_ by Ni-NTA and ion exchange chromatography yielded two prominent protein fragments, both corresponding to TET1_CD_ (Supplementary Fig. 2). Mass spectrometry identified the smaller ∼75 kDa fragment as an N-terminal truncation of ∼240 amino acids, including the conserved Cys-rich domain, which was shown to be essential for the catalytic activity^32^. To test the catalytic activity of this His6–TET1_CD_ preparation (Fig. 2b), we *in vitro* methylated highly pure plasmid DNA using the M.SssI CpG methyltransferase to completion (200 pmol mCpG sites per μg DNA), reacted this substrate DNA (200 ng, 40 pmol mCpGs) with purified His6–TET1_CD_ (500 ng, 6 pmol) at 37 °C for 1 h and detected the cytosine modifications generated by immunoblot analysis with antibodies against 5mC, 5hmC, 5fC and 5caC. All detectable 5mC was fully oxidized to 5hmC, 5fC or 5caC under the reaction conditions mentioned. His6–TET1_CD_ thus carried out all predicted 5mC oxidation steps *in vitro*, whereby the conversion of 5mC to 5hmC appeared to be the most efficient step (Fig. 2b).

We next used separately purified TET and TDG proteins to reconstitute the 5mC release. To allow for preformation of the TET–TDG complex, we mixed His6–TET1_CD_ with His6–TDG (Supplementary Fig. 2) at a ratio of 100:50 nM (most active ratio by titration), respectively, before addition of DNA substrates. A twofold molar excess of TET–TDG (50 nM complex) over substrate DNA (25 nM) and an incubation of 60 min at 37 °C resulted in efficient release of both 5mC and 5hmC (Fig. 2c). Notably, 5mC was nearly as efficiently excised as 5hmC, which, given the single turnover setup in this assay, indicated that the oxidation of 5hmC by TET1_CD_ was rate limiting in this assay. 5caC served as a control for TDG activity and was processed most efficiently, as expected.

Together, these results establish that TET1_CD_ and TDG activities can be combined to act in concert to efficiently excise 5mC from DNA, thereby generating alkaline labile AP-sites in DNA.

### TET stabilizes TDG activity

**Figure 3:**
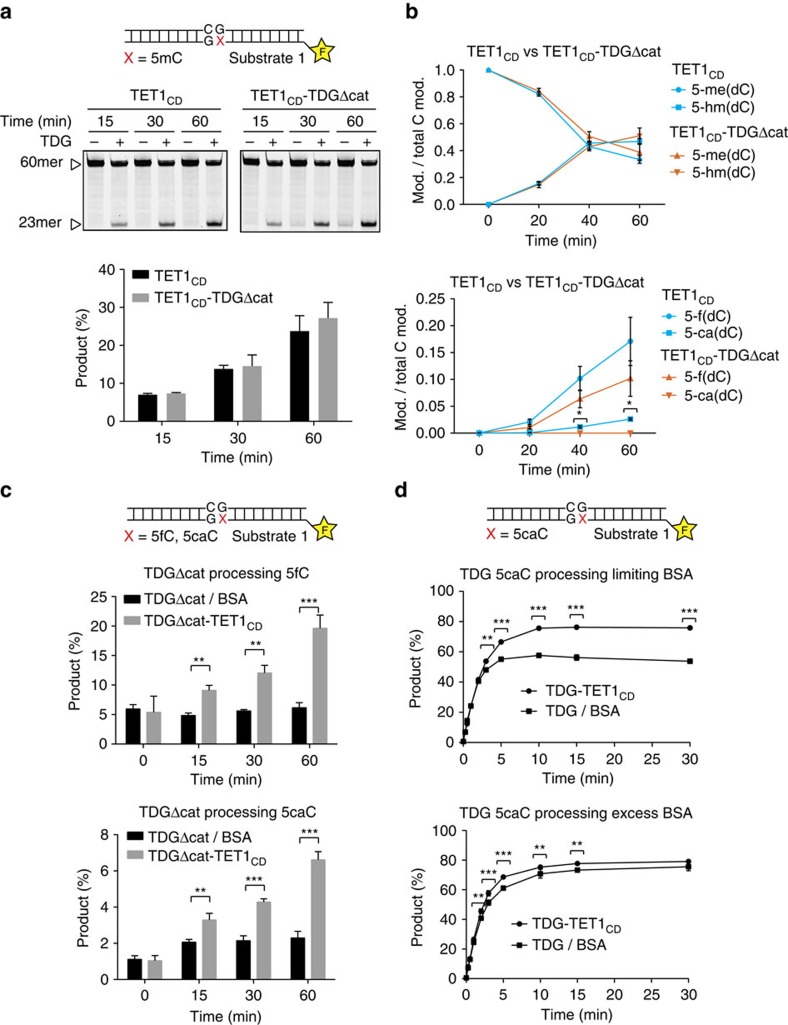
TET1_CD_ stabilizes TDG activity. (**a**) Stimulatory effect of His6–TDGΔcat on His6–TET1_CD_ examined by base release assay. His6–TET1_CD_ (50 nM) or His6–TET1_CD_–His6–TDGΔcat (25 nM) complex were incubated with DNA substrate (25 nM) containing a G·5mC base pair for the indicated time. Recovered DNA was then assayed by a base release assay using His6–TDG (250 nM) to monitor the presence of oxidized 5mC species. (**b**) LC–MS/MS analysis of plasmid oxidation assays using His6–TET1_CD_ or TET1_CD_–His6–TDGΔcat. *In-vitro*-methylated pUC19 plasmid DNA (660 nM) was treated with either TET1_CD_ (100 nM) or a preassembled TET1_CD_–TDGΔcat complex for the indicated time. DNA was analysed by LC–MS/MS; shown are normalized mean values (mod/total C mod). (**c**) Activity of His6–TDGΔcat on 5fC and 5caC in the presence or the absence of His6–TET1_CD_. His6–TDGΔcat–BSA (50 nM) or His6–TDGΔcat–His6–TET1_CD_ (50 nM) were incubated with DNA substrate (25 nM) containing a G·5fC or G·5caC at 37 °C, reactions were stopped by the addition of NaOH at indicated time and analysed by denaturing gel electrophoresis. (**d**) The effect of His6–TET1_CD_ on His6–TDG catalysis assessed in base release assays. The time-dependent generation of AP-sites was measured after reaction of a 60mer substrate containing a single G·5caC (25 nM) base pair with a preassembled His6–TDG–His6–TET1_CD_ (25 nM) or His6–TDG–BSA (25 nM) complex in the presence (lower panel) or absence (upper panel) of a 60-fold molar excess of BSA. Reactions were stopped by the addition of NaOH after the indicated time and analysed using denaturing gel electrophoresis and fluorescent scanning. Shown are mean values with s.d. *P*-values were calculated by the Student's *t*-tests (*P<0.05, **P<0.01 and ***P<0.001).

To address whether TET1 and TDG cooperate at the level of their catalytic activities, we examined the effect of TDG on the efficiency of 5mC oxidation by TET1_CD_. In this setup, we used purified, catalysis-deficient TDG, to limit excision of TET-generated 5fC and 5caC. We pre-incubated His6–TET1_CD_ (50 nM) with or without His6–TDGΔcat (25 nM), added 5mC substrate (25 nM), stopped the reactions at different time points and monitored the presence of 5fC and 5caC in the recovered DNA by digestion with purified active TDG (250 nM). The presence of TDGΔcat had a minor effect on 5mC oxidation by His6–TET_CD_ (Fig. 3a). This result was corroborated in a methylated plasmid oxidation assay analysed by quantitative liquid chromatography tandem mass spectrometry (LC–MS/MS). In this assay, we did however observe a slightly reduced conversion of 5hmC to 5fC and 5caC in the presence of TDGΔcat (Fig. 3b). The reduced 5fC levels and the lack of detectable 5caC in the reactions with TDGΔcat probably reflect the residual activity of the TDG catalytic mutant towards these substrates, in particular in the presence of TET1_CD_ (Fig. 3c)^31^. The catalytic dead TDG may also mask 5fC and prevent further oxidation by TET1_CD_. These results show that under single turnover conditions, purified His6–TET1_CD_ can efficiently oxidize 5mC to generate 5fC/5caC, irrespective of whether TDG is present or not, although its efficiency in 5hmC oxidation may be reduced under multiple turnover conditions.

*Vice versa*, the presence of a twofold molar excess of TET1_CD_ had a positive effect on TDG activity on 5fC and 5caC excision when compared with BSA added to the same molarity. This was most notable in reactions with TDGΔcat where the glycosylase activity is rate limiting (Fig. 3c). Under these conditions, excision of both 5fC and 5caC was significantly increased in the presence of TET1_CD_, in particular after prolonged incubation, indicating that the interaction of TET1_CD_ with TDGΔcat stabilizes TDG activity. Notably, the stimulation observed in 5fC excision may in part be due to the conversion of 5fC to 5caC in the presence of TET1_CD_ and reflect the activity of TET1_CD_–TDGΔcat on 5caC. TET1_CD_ also stabilized the fully active wild-type TDG; a twofold molar excess of TET1_CD_ significantly enhanced TDG-dependent excision of 5caC (Fig. 3d upper panel) and T (Supplementary Fig. 3b). As a large molar excess of BSA was required to achieve a similar stabilizing effect on TDG (Fig. 3d lower panel), we conclude that the TET1 effect is due to its specific interaction with TDG.

### Reconstitution of TET-BER-mediated DNA demethylation

**Figure 4:**
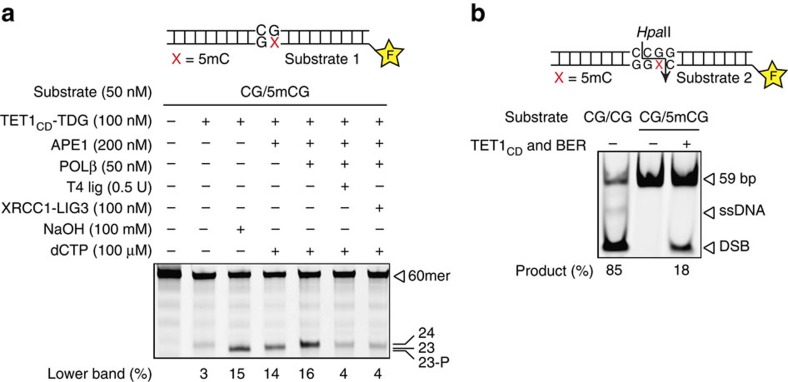
Full reconstitution of TET–TDG–BER-mediated DNA demethylation. (**a**) Intermediate steps of the oxidative DNA demethylation reaction were reconstituted and visualized by denaturing gel electrophoresis. Labelled 60mer substrate DNA containing one G·5mC base pair was incubated sequentially with TET–TDG–BER enzymes at concentrations indicated. Reaction products were separated by denaturing gel electrophoresis and visualized by fluorescent scanning; sizes of the 60mer substrate DNA and reaction products are indicated. (**b**) Complete DNA demethylation by the reconstituted TET–TDG–BER system analysed by the generation of a HpaII-sensitive restriction site. Reconstituted DNA demethylation was done with a 5′-labelled 59-bp substrate containing one G·5mC base pair within a HpaII recognition site (CCGG). Recovered DNA was digested with methylation-sensitive HpaII endonuclease and analysed by native PAGE; positions of the 59-bp substrate DNA and product fragment are indicated.

TET1–TDG-mediated active DNA demethylation implicates the engagement of BER in the restoration of the unmethylated DNA sequence following 5fC or 5caC excision. To formally proof the functionality of such a pathway and to provide a tool to investigate its mechanistic features, we reconstituted the entire process of active DNA demethylation with defined components. In addition to TET1_CD_ and TDG, we purified to near homogeneity the enzymes of the core BER pathway^33^, APE1, POLβ and XRCC1-LIG3 (Supplementary Fig. 2). Using a 60-bp substrate containing a single 5mC, we first performed demethylation in step-by-step reactions to monitor the DNA intermediates generated. The combined action of His6–TET1_CD_ and His6–TDG generated an AP-site cleavable either chemically by NaOH or enzymatically by APE1, to generate a 23-nt oligonucleotide with or without a 3′-phosphate, respectively (Fig. 4a lanes 3 and 4). Following strand incision by APE1, POLβ was able to insert a deoxycytidine monophosphate, thus generating a 24-nt oligonucleotide as the main product (Fig. 4a lane 5). Addition of either T4 ligase or an XRCC1–LIG3 complex then efficiently ligated the nicked intermediate, restoring a continuous 60mer DNA fragment (Fig. 4a lanes 6 and 7). The nearly complete re-ligation confirmed the efficient removal of the 5′-dRP remains of the cleaved AP-site by POLβ. These results establish that TET1 and TDG convert 5mC to DNA repair intermediates amenable to processing by the core BER system.

To test the accuracy of the reconstituted DNA demethylation process, we performed the reaction with a 59-bp DNA substrate presenting a hemimethylated CpG dinucleotide within a recognition site for the HpaII endonuclease (CCGG) (Fig. 4b). Owing to its methylation sensitivity, HpaII will not be able to cleave this substrate unless it undergoes successful and complete demethylation. We subjected the hemimethylated substrate to demethylation by the reconstituted TET–TDG–BER system and examined the generation of a cleavable restriction site by HpaII digestion (Fig. 4b). As expected, the asymmetrically methylated substrate was fully resistant to HpaII cleavage (Fig. 4b lane 2), as were substrates carrying the predicted intermediates 5hmC, 5fC and 5caC (Supplementary Fig. 4). Incubation in the presence of the reconstituted DNA demethylation system, however, generated HpaII-digestible DNA products, indicating that the methylated DNA fragment was converted into an intact unmethylated fragment (Fig. 4b lane 3). Together, these results proof that TET1–TDG-mediated oxidation and excision of 5mC generates intermediates for BER, which then acts to efficiently restore the original DNA sequence in an unmethylated configuration.

### Coordinated TET-TDG-BER action avoids DSB formation

**Figure 5:**
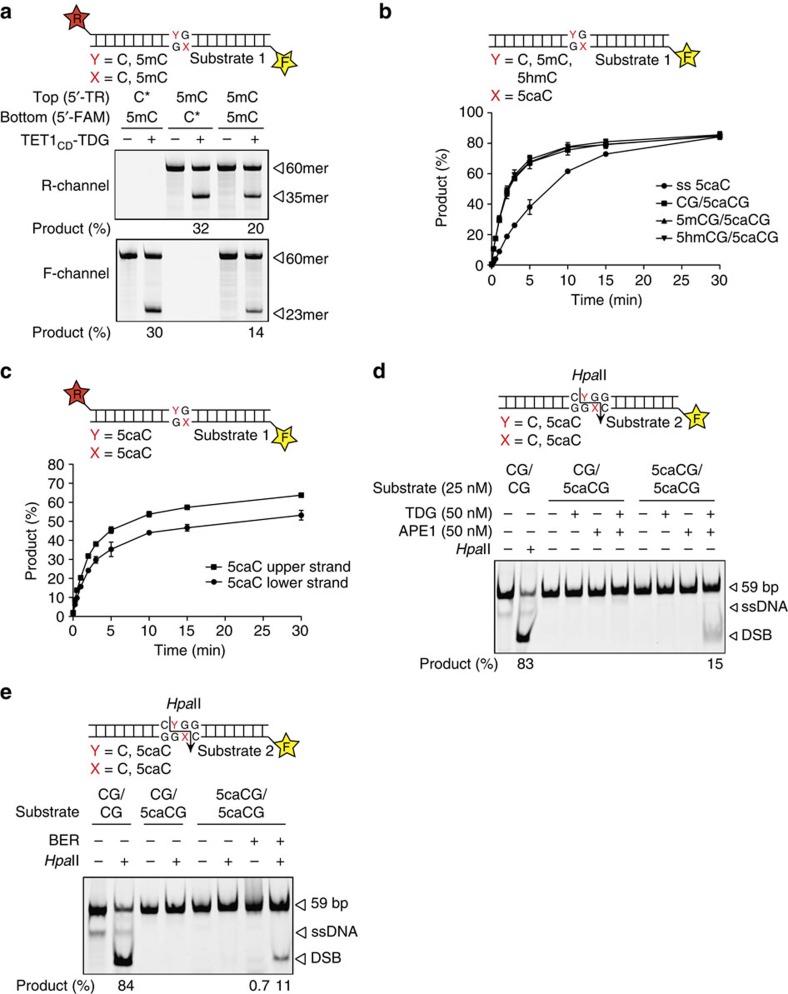
Processing of differentially modified CpGs by TET1_CD_–TDG or TDG. (**a**) Base release from fully methylated CpGs by His6–TET1_CD_–His6–TDG. His6–TET1_CD_–His6–TDG (50 nM) was incubated with labelled 60mer substrates (25 nM) containing a single 5mC modification on the fluorescent labelled top (5′-Texas Red, T) or bottom strand (5′-fluorescein, F) or a fully methylated CpG with labels on both strands. Product formation was monitored and quantified by denaturing gel electrophoresis and fluorescent scanning (Texas Red, R-channel and fluorescein, F-channel); positions of the 60mer substrate DNA and the resulting base incision products of both strands are indicated. *Unlabelled DNA strand. (**b**) Release of 5caC from differentially modified CpGs by His6–TDG. 60mer DNA substrates (25 nM) containing 5caC opposite C, 5mC or 5hmC in a CpG dinucleotide or in single-stranded (ss) DNA were incubated with His6–TDG (25 nM) and analysed by denaturing gel electrophoresis. Shown are mean percentages of product formation with s.d. (*n*=3). (**c**) 5caC release from a symmetrically modified CpG dinucleotide by His6–TDG. A substrate (25 nM) containing 5caC on both strands within a CpG dinucleotide and labels of both strands was incubated with His6–TDG (25 nM) for indicated time, analysed by denaturing gel electrophoresis and visualized by fluorescent scanning of both labels. Shown are mean percentages of product formation with s.d. (*n*=3). (**d**) TDG and APE1 only generate DNA DSBs at symmetrically modified CpGs. Base release assay using TDG and APE1 on a labelled 59-bp substrate containing either a single 5caC or a symmetrically 5caC-modified base pair within a HpaII recognition site (CCGG). Reactions were analysed by native PAGE. Substrate DNA and product fragment are indicated. (**e**) Full reconstitution of TDG–BER on a symmetrically modified 5caC substrate. A labelled 59-bp substrate (25 nM) containing a symmetrically 5caC-modified base pair within a HpaII recognition site (CCGG) was incubated with TDG (40 nM) and BER factors (200 nM APE1, 40 nM POLβ and 40 nM XRCC1-LIG3). Recovered DNA was digested with HpaII endonuclease and analysed by native PAGE. Substrate DNA and product fragments are indicated; ssDNA, free single-stranded DNA.

CpGs in mammalian DNA are mostly symmetrically methylated, generating a potential conflict for excision-repair-mediated DNA demethylation; that is, once started, a DNA demethylation event in one DNA strand would have to be completed before another event starts at the symmetrically opposite 5mC, which would otherwise lead to the formation of a DNA double-strand break (DSB). We therefore asked whether in a symmetrically methylated CpG dinucleotide, demethylation events would generate DSBs or be confined to one strand at a time. For this purpose, we generated three 60-bp DNA substrates with either a fluorescein-labelled bottom strand containing a single 5mC, a Texas Red-labelled top strand containing a single 5mC or both strands labelled and presenting a symmetrically methylated CpG (Fig. 5a). Incubation of all these substrates (25 nM) with a twofold molar excess of TET1_CD_–TDG produced a solid 5mC release from both the bottom and the top strands, irrespective of whether the CpG was hemi- or symmetrically methylated (Fig. 5a). Activities on the top and bottom strands in hemimethylated substrates were similar, indicating the absence of sequence context effects in this setup (Fig. 5a lanes 2 and 4). Notably, the same reaction conditions applied to the substrate with 5mC modifications on both strands produced approximately half the amount of incised product on each DNA strand with the total activity remaining constant (Fig. 5a lane 6). These results show that TET1_CD_–TDG can act on both strands on a substrate containing a symmetrically methylated CpG and suggested that it does so in a sequential manner affecting only one strand at a time.

To further investigate the demethylation events at symmetrically modified CpGs, we separated TDG from TET1 activities and measured the kinetics of 5caC processing in the context of potentially arising DNA demethylation intermediates. Using equimolar substrate and enzyme concentrations^34^ (25 nM), we evaluated substrates containing a 5caC on the labelled DNA strand opposite an unmodified C, a 5mC or a 5hmC within the same CpG (Fig. 5b). Under the conditions applied, both initial rate and overall 5caC excision by TDG was not notably affected by the modification status of the symmetrically opposite C (Fig. 5b). 5caC was processed with appreciable efficiency even in single-stranded DNA, corroborating the high affinity of TDG for this substrate. The situation when 5caC arises in both strands simultaneously is of particular interest, as it raises the possibility that TDG-initiated BER will induce DNA DSBs. We thus evaluated the behaviour of TDG in such a context, monitoring the release of 5caC from both strands in a time-course base release assay with a substrate (25 nM) carrying labels on both strands. Similar to the activity of TET1–TDG on 5mC, TDG alone acted evenly on both strands carrying the 5caC (Fig. 5c). The resulting plateau of single-strand incision at ∼50% indicated that the processing of one DNA strand by TDG largely inhibited base release from the other strand. This is a probable consequence of TDGs tight interaction with AP-sites^31^,^35^, the coordinated dissociation of which^36^,^37^ may favour completion of the repair process to initiation of an additional repair event at the opposite strand. Only after prolonged incubation (30 min) this plateau increased above 50% for one DNA strand, indicating some turnover of TDG. To test whether the spontaneous turnover of TDG could eventually generate symmetrical AP-sites and potentially cause DSBs, we included APE1 (50 nM) in the assay (Fig. 5d). The combined action of TDG and APE1 indeed produced a notable fraction of DNA DSBs (15%). We therefore asked whether repair of a symmetrical demethylation intermediate is at all possible and can be achieved without the generation of DSBs. We thus used a substrate with a symmetrically 5caC-modified HpaII site in a reconstituted TDG–BER assay and analysed the generation of cleavable HpaII sites. Both, hemi- and symmetrically 5caC-modified substrates were fully resistant to HpaII cleavage (Fig. 5e lane 4 and 6). However, incubation of the symmetrically 5caC-modified DNA (25 nM) with the TDG–BER system (40 nM TDG, 200 nM APE1, 40 nM POLβ and 40 nM XRCC1-LIG3) generated an appreciable amount of HpaII cleavable product, indicating that the 5caCs in both DNA strands were replaced with unmodified Cs (Fig. 5e lane 8). Notably, this process of symmetrical repair, which ultimately requires the breaking of both DNA strands did not lead to an accumulation of DSBs (<1%) (Fig. 5e lane 7), suggesting that in the presence of all repair factors repair events at both strands proceed preferentially in a sequential manner.

From these results, we conclude that DNA demethylation *in vitro* has no apparent strand and hence sequence-context preference. TET1–TDG is capable of initiating active DNA demethylation in both strands of a fully methylated CpG. Once initiated in one strand, however, BER is completed before it restarts on the other strand, indicating that demethylation of symmetrically methylated CpGs occurs in a sequential manner.

### DNA demethylation inhibits G·T repair at methylated CpGs

**Figure 6:**
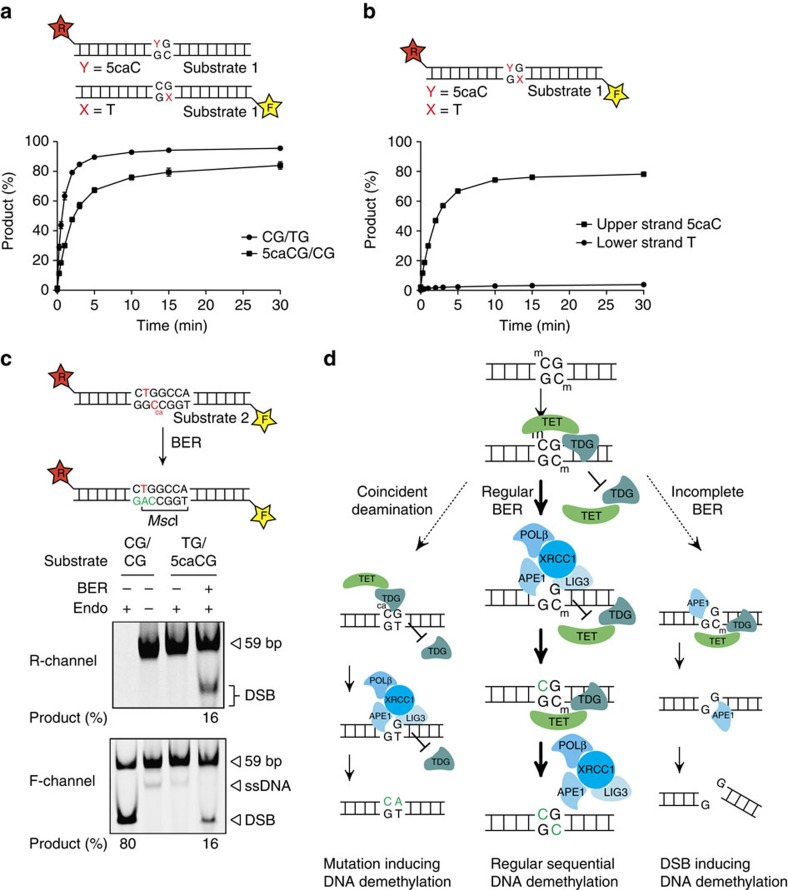
DNA demethylation blocks G·T repair and can induce mutations. (**a**) Enzymatic activity of TDG on G·5caC- and G·T-containing substrates. Release of 5caC and T by His6–TDG (25 nM) was monitored over time on 5′-labelled 60-bp substrates (25 nM) containing either a G·5caC or G·T base pair. Reactions were stopped at indicated time, separated by denaturing gel electrophoresis, visualized with fluorescent scanning and quantified. Shown are mean percentages of product formation with s.d. (*n*=3) (**b**) Base release from a substrate containing a G·5caC next to a G·T mismatch. Substrate preference of TDG (25 nM) was evaluated on a 59-bp DNA fragment (25 nM) containing 5caC on the labelled top strand (5′-Texas Red) and T on the labelled bottom strand (5′-fluorescein) within the same CpG context as illustrated. Reactions were stopped after indicated time, separated by denaturing gel electrophoresis, and both strands visualized by fluorescent scanning and quantified. Shown are mean percentages of product formation with s.d. (*n*=3). (**c**) Full reconstitution of TDG–BER on a G·5caC/G·T-containing substrate. A labelled 59-bp substrate containing a G·5caC next to a G·T mismatch was incubated with His6–TDG and BER factors. Correct repair of the 5caC and the introduction of an A opposite of T was monitored by MscI digestion and analysed by native PAGE and fluorescent scanning. Unmodified (CG/CG) substrate DNA digested with HpaII was used as size marker; positions of the substrate DNA and product fragments are indicated. ssDNA, free single-stranded DNA. (**d**) Mechanistic model of TET–TDG–BER-mediated DNA demethylation. In the presence of all the necessary factors, DNA demethylation at fully methylated CpGs occurs in a coordinated and sequential manner to correctly re-establish the unmodified state (regular BER). Lack of coordination, for example, in the absence of downstream BER factors, repair-mediated DNA demethylation can lead to the induction of DNA DSBs (incomplete BER). Coincident oxidation and hydrolytic deamination at fully methylated CpG sites can lead to increased C to T transitions caused by the sequential repair mechanism (coincident deamination).

Another issue of BER-mediated demethylation at symmetrically methylated CpGs is the potential collision with 5mC deamination. 5mC in genomic DNA is susceptible to spontaneous hydrolytic deamination^38^, generating a thymine paired with a guanine. Such G·T mismatches are recognized and excised also by TDG. Enzymatic deamination coupled to BER has also been considered as a mechanism of active DNA demethylation^7^,^24^,^39^,^40^; it would replace a 5mC with an unmodified C through a mutagenic intermediate. To investigate potential interferences between deamination and oxidation-induced DNA demethylation pathways, we evaluated G·T and G·5caC processing efficiencies in kinetic base release assays, using equimolar substrate/enzyme (His6–TDG) concentrations (25 nM). When provided on separate DNA molecules, TDG processed the G·T mismatch more efficiently than the 5caC substrate (Fig. 6a), showing that the mismatch is a preferred substrate as reported previously^16^. In a substrate where the G·5caC modification is next to a G·T mismatch within the same CpG dinucleotide, reflecting a spontaneous deamination event on one strand while the other is being actively demethylated, TDG processes almost exclusively the 5caC, leaving the G·T mismatch untouched (Fig. 6b). The processing rate of 5caC was largely unaffected by the presence of the G·T mismatch, indicating that in this configuration 5caC is clearly the preferred substrate. The result was essentially the same when the modifications were inversed within the same double-stranded substrate (Supplementary Fig. 5), thus excluding DNA strand or sequence-context effects as an explanation for the preference for 5caC.

This strong preference of TDG for the non-mutagenic 5caC next to a pre-mutagenic G·T mismatch implies that TET–TDG-mediated active DNA demethylation has a potential to mutate CpG dinucleotides if it coincides with a deamination event. To test this possibility, we used our fully reconstituted BER setup on a 59-bp substrate containing a G·5caC next to a G·T mismatch within an MscI recognition site and analysed the generation of mutant demethylation products by endonuclease digestion. 5caC-directed sequential BER of this substrate would generate C to T mutations and thus create an MscI restriction site if two or more nucleotides were incorporated during the DNA resynthesis step (Fig. 6c). In the absence of the TDG–BER machinery, no MscI cleavage products were detectable (Fig. 6c lane 3). However, full reconstitution of TDG–BER generated a cleavable product, indicating that the 5caC was correctly replaced with a C but an A was incorporated opposite of T, thus manifesting the C to T transition and a loss of a CpG dinucleotide (Fig. 6c lane 4).

## Discussion

Recent research on active DNA demethylation points towards a mechanism involving TET proteins and the DNA glycosylase TDG as well^12^,^13^,^14^,^16^,^17^. A current model suggests that DNA demethylation through this pathway occurs in a stepwise manner via TET-catalysed oxidation of 5mC to 5fC and 5caC, which are then excised by TDG-dependent BER to restore an unmethylated DNA sequence. Despite the plausibility of this pathway, experimental evidence that directly links TET activity with TDG and BER is missing and fundamental mechanistic questions have not been addressed. The data presented here provide strong evidence for a coupling of 5mC oxidation and TDG-initiated BER in a cascade of enzymatic reactions that productively demethylates DNA. *In vitro* reconstitution of the active demethylation of symmetrically modified CpGs revealed a mechanism that is intrinsically coordinated to operate sequentially on both strands. Although this prevents the formation of DNA DSBs, and hence genomic instability, the process can be mutagenic if 5mC deamination and oxidative demethylation events coincide on opposite strands in a CpG dinucleotide.

In line with co-localization studies^41^, our work provides biochemical evidence for a direct and specific physical interaction of TET1 with TDG, implicating a link between 5mC oxidation and base excision. This interaction allowed us to enrich a functional TET1–TDG complex from *E. coli* lysates that was highly active and capable of removing 5mC from a synthetic DNA substrate. In contrast to previous studies, showing 5mC conversion by TET and base excision by TDG in separate assays^12^,^13^,^16^, our data demonstrate a concerted action of both enzymes in 5mC oxidation and excision.

The relative high abundance of 5hmC in cells compared with 5fC and 5caC^13^,^42^ suggests that 5mC oxidation by TET enzymes is tightly regulated. A straightforward explanation could be that the rate of the oxidation of 5mC to 5hmC by TET enzymes is higher than that of the subsequent oxidations of 5hmC or 5fC, which may require stimulation by the presence of additional factors, such as the TDG^13^,^43^ and/or Gadd45 (refs 44, 45). We examined this possibility but did not measure a stimulatory effect of TDG on TET1_CD_ catalysis at any step of oxidation, neither did we observe such an effect for Gadd45a added to TET1_CD_–TDG (Supplementary Fig. 3a). These experiments were done with TET1_CD_, however, leaving the possibility that the missing N terminus with its zinc finger CXXC domain may provide such regulatory function. Additional work is needed to address the mechanism of TET1 regulation, that is, to identify the factors determining the patterning of genomic 5hmC, 5fC and 5caC generation. The reconstituted demethylation assay presented here will be instrumental in this endeavor.

The engagement of a DNA glycosylase in active DNA demethylation inevitably generates a need for AP-sites repair. Evidence supporting an involvement of the BER pathway has been reported for primordial germ cells, where an increase of DNA single-strand breaks and BER activity was linked to active global DNA demethylation^46^ and, in a more recent study, where various BER proteins were found to co-precipitate with overexpressed TET1 (ref. 41). With the successful reconstitution of TET1–TDG–BER-mediated DNA demethylation, we provide the first evidence for a physical and functional coupling of these factors in the oxidation and excision of 5mC and the resynthesis of an unmethylated C. Although such BER-mediated DNA demethylation seems mechanistically straightforward, it raises concerns regarding potential adverse effects on genome stability, in particular where the density of CpGs undergoing demethylation is high and excessive formation of DNA strand breaks might occur. It is therefore fair to assume that active demethylation in cells is a highly orchestrated process, controlled through regulatory mechanisms also involving posttranslational modifications^18^,^36^,^37^. Our *in vitro* DNA demethylation system does not recapitulate regulatory actions of this kind but it does inform on intrinsic features of the mechanism regarding the potential of DNA DSB formation and the handling of complex substrates.

A distributive mode of action of TET proteins, for instance, would produce a variety of demethylation intermediates with 5caC placed opposite from 5mC, 5hmC or C within CpG dinucleotides, the precise configuration of which may then determine the efficiency of initiation of BER. However, this seems an unlikely regulatory concept, as TDG processed 5caC with high efficiency irrespective of the opposite C modification. Yet, our experiments indicate that although the TET–TDG demethylase is capable of acting on both strands at symmetrically modified CpGs, it does so in a sequential manner without producing DNA DSBs. Even with substrates containing the efficiently processed 5caC in both strands, TDG-mediated BER did not generate detectable DNA DSBs and this was not due to a preference of TDG for one strand in particular. In the case of an occurrence of symmetrical substrates within CpG dinucleotides, such as during symmetrical DNA demethylation, the high-affinity binding of TDG to AP-sites^31^,^35^ may constitute an important protective mechanism; not only will it provide an opportunity to coordinate AP-site repair but also protect the opposite strand from being processed at the same time. The importance of coupling base excision with the BER process in this delicate situation is highlighted by the observation that in the absence of POLβ and XRCC1-LIG3, TDG and APE1 generated an appreciable amount of DSBs in symmetrically modified substrates (Fig. 5d). We therefore argue that BER in the context of active DNA demethylation occurs in a processive manner, where the initially attacked strand is fully repaired before processing of the opposite strand (Fig. 6d). This may explain how the replacement of symmetrical 5mC with unmodified C can occur without destabilizing the genome.

Another situation that may arise is the coincident deamination and oxidation of opposed 5mCs in CpG dinucleotides. Methylated CpGs are well known for their increased mutability, which is, to a large extent, due to the higher rate of hydrolytic deamination of methylated cytosines compared with unmethylated cytosines^47^. Such deamination will generate premutagenic G·T mispairs within methylated CpG dinucleotides^38^. This observation alone does not adequately explain the relatively high C to T mutation rates at such sites, as cells have efficient mechanisms in place to repair these G·T mismatches, for example, TDG- or MBD4-mediated BER^18^. Our data on G·T versus 5caC repair in CpG dinucleotides provide a plausible explanation for how G·T mismatches might escape correction and turn into mutations. Although, consistent with previous observations^16^, TDG processed the G·T mismatch with higher efficiency than 5caC when the two lesions were analysed separately, 5caC was processed with a striking preference when both were present within the same CpG, reflecting a situation where deamination occurs at a site undergoing active demethylation (Fig. 6d). This strong preference for the perfectly base-paired 5caC is consistent with a high-affinity binding of TDG to 5caC as implicated by the uniquely specific active site contacts it establishes with this base^48^,^49^. The sequential repair of both lesions, which helps avoid DSB formation, then turns into a disadvantage in this particular situation. The initiation of repair at the 5caC would mask a nearby G·T mismatch for repair and fix the C to T mutation within the CpG dinucleotide whenever the resynthesis step of BER incorporates two or more nucleotides (Fig. 6d).

In conclusion, our data provide proof of functionality of an active DNA demethylation pathway based on the coupled oxidation and excision repair of 5mC; they provide insight into how intrinsic features of the mechanism allow demethylation of symmetrically methylated CpGs without the formation of DNA DSBs and how it may contribute to C to T mutagenesis within methylated CpG dinucleotides. Having a fully reconstituted DNA demethylation process established will allow future investigations into the detailed mechanism of the process, including the important aspect of TET regulation.

## Methods

### Bacterial expression vectors

The plasmids for the expression of TDG (GI: 37589917) (pTG-mTDGa.0, pET28-mTDGa.0 and pET28-mTDGa.1), TET1 (GI: 568968019) (pCDF-mTET1), TET1 catalytic domain (aa 1367–2057) (pCDF-His-mTET1CD and pCDF-His-mTET1CDΔcat), TET1 N terminus (aa 301–1366) (pACYC-mTET1-N), APE1 (GI:18375501) (pPRS125 and pEThis-APE1.0), POLβ (GI:4505931) (pPRS112 and pQE30-6HIS-Polβ), XRCC1 (GI:190684675) (pET-XRCC1) and LIG3 (GI:73747829) (pGEX4T-Lig3) were assembled by standard cloning methods based on PCR amplification with adaptor-oligonucleotides providing suitable restriction sites.

### Antibodies

The following antibodies were used: TDG, rabbit polyclonal antibody 141, 1:20,000; TET1_CD_, rabbit polyclonal α-TET1 antibody (Millipore, catalogue number 09-872), 1:5,000; TET1-N, rabbit polyclonal α-TET1 antibody (Genetex, catalogue number GTX124207), 1:10,000; 5mC, mouse monoclonal α-5mC antibody (Diagenode, catalogue number C15200081), 1:250; 5hmC, rabbit polyclonal α-5hmC (Active motif, catalogue number 39769), 1:20,000; 5fC rabbit polyclonal α-5fC (Active motif, catalogue number 61223), 1:2,500; 5caC, rabbit polyclonal α-5caC (Active motif, catalogue number 61225), 1:2,000.

### 5-Carboxyethyl-N4-benzoyl-dC CE phosphoramidite

The 5caC phosphoramidite (5-carboxyethyl-N4-benzoyl-dC CE) was synthesized in collaboration with Glen Research (USA).

### Oligonucleotides

60mer (Substrate 1 and 3) or 59mer (Substrate 2) double-stranded oligonucleotide substrates containing different modifications were prepared by annealing of two complementary oligonucleotides synthesized by Adam Robertson or Microsynth (Switzerland). The upper strand was either unlabelled or carried a 5′-Texas Red label, whereas the lower strand was unlabelled or carried a 5′-fluorescein label. Substrate 1 (standard) upper strand 5′-TAGACATTGCCCTCGAGGTACCATGGATCCGATGTXGACCTCAAACCTAGACGAATTCCG-3′ where X=C, T, 5mC, 5hmC or 5caC. Substrate 1 lower strand strand 5′-CGGAATTCGTCTAGGTTTGAGGTXGACATCGGATCCATGGTACCTCGAGGGCAATGTCTA-3′, where X=T, 5mC, 5hmC or 5caC. Substrate 2 upper strand 5′-TAGACATTGCCCTCGACGACCCGCCGCCGCGCXGGCCACCCGCACCTAGACGAATTCCG-3′ where X=C, T, 5mC, 5hmC or 5caC. Substrate 2 lower strand 5′-CGGAATTCGTCTAGGTGCGGGTGGCXGGCGCGGCGGCGGGTCGTCGAGGGCAATGTCTA-3′ where X=5mC, 5hmC or 5caC. Substrate 3 upper strand 5′-TAGACATTGCCCTCGACGGTGCCCTCXGGGCCGCGCGTCGCGCTCCCTAGACGAATTCCG-3′ where X=C. Substrate 3 lower strand 5′-CGGAATTCGTCTAGGGAGCGCGACGCGCGGCCXGGAGGGCACCGTCGAGGGCAATGTCTA-3′ where X=5mC or 5hmC.

### Recombinant protein expression

The expression vectors were introduced into *E. coli* BL21(DE3) cells by electroporation. Overnight precultures were diluted with fresh prewarmed LB broth medium and grown at 30 °C to an OD_600_ level of 0.6–0.8. Cultures were grown under selective pressure using respective antibiotics at concentrations of either 100 mg l^−1^ (ampicillin) or 50 mg l^−1^ (kanamycin and streptomycin) for single plasmid expressions and half the concentration of each antibiotic when co-expressing two plasmids. Protein expression was induced using the following conditions: TET1 (250 μM isopropyl-β-D-thiogalactoside (IPTG), 25 °C for 3 h), TET1–TDG (250 μM IPTG, 25 °C for 3 h), TDG (250 μM IPTG, 15 °C for 16 h), APE1 (500 μM IPTG, 25 °C for 6 h), POLβ (500 μM IPTG, 25 °C for 3.5 h), and LIG3 and XRCC1 were co-expressed (250 μM IPTG, 25 °C for 4 h). Finally, cells were harvested by centrifugation and soluble protein fractions were extracted by sonication (Bioruptor, Diagenode) or homogenization (Emulsiflex C-3, Avestin) in lysis buffer (50 mM Na-phosphate buffer pH 7.5, 300 mM NaCl, 20% glycerol, 0.1% Tween-20, 1 mM dithiothreitol (DTT), 1 mM phenylmethylsulfonyl fluoride (PMSF)), if not stated otherwise. Crude lysates were then cleared by centrifugation with >30,000 *g* at 4 °C for 60 min.

### Protein purification

For TET1_CD_ purification, the cleared lysate was loaded onto a 1-ml HisTrap FF crude column (GE Healthcare, Germany), bound protein was eluted with 400 mM imidazole and relevant fractions dialysed against CIEX buffer (50 mM HEPES pH 7.2, 25 mM NaCl, 20% glycerol, 5 mM DTT and 0.1 mM PMSF). Dialysed fractions were then loaded onto a 1-ml Resource S column (GE Healthcare) and bound protein was eluted with a linear salt gradient of 25 mM–1 M NaCl and purest fractions finally dialysed against storage buffer (50 mM HEPES pH 7.2, 100 mM NaCl, 20% glycerol and 5 mM DTT), frozen on dry ice and stored at −80 °C.

BER proteins were purified as followed; in brief, APE1 and POLβ were purified by Ni-NTA affinity and ion exchange chromatography^34^,^50^, and TDG was purified by Ni-NTA affinity, heparin affinity and ion exchange chromatography as follows^51^. Briefly, *E. coli* lysates were prepared by sonication (12 times for 30 s on ice with intermittent chilling) and clarified by centrifugation (Sorvall SS34, 18,000 r.p.m., 4 °C) at 4 °C. The supernatant was applied to a disposable column packed with 1.5 ml preequilibrated Ni-NTA agarose (Qiagen) at a flow rate of 15 ml h^−1^. After washing with 120 ml sonication buffer (50 mM Na-phosphate pH 8.0, 750 mM NaCl, 20% glycerol, 1 mM imidazole, 10 mM β-mercaptoethanol and 1 mM PMSF), bound proteins were eluted with 10 ml sonication buffer containing 500 mM imidazole and dialysed against buffer H50 (50 mM Na-phosphate pH 8.0, 50 mM NaCl, 20% glycerol, 10 mM β-mercaptoethanol, 1 mM PMSF). After loading the dialysed fraction onto a 5-ml HiTrap Heparin HP column (GE Healthcare) at a flow rate of 1 ml min^−1^ and washing with 10 ml H50, bound protein was eluted with a liner gradient of 50-800 mM NaCl in 50 ml. Purest fractions were pooled, dialysed against buffer Q20 (50 mM Na-phosphate pH 8.5, 20 mM NaCl, 10% glycerol, 10 mM β-mercaptoethanol, 1 mM PMSF) and loaded on a 1-ml HiTrap Q HP at a flow rate of 1 ml min^−1^. After washing with 10 ml Q20 buffer, bound proteins were eluted with a linear gradient of 20–500 mM NaCl in 15 ml. The fractions containing TDG with >98% homogeneity were pooled, dialysed against storage buffer (50 mM Na-phosphate pH 8.0, 50 mM NaCl, 10% glycerol, 10 mM β-mercaptoethanol, 1 mM PMSF), frozen in liquid nitrogen and stored at −80 °C. LIG3 and XRCC1 were purified as a complex by Ni-NTA (HisTrap HP; 50 mM Na-phosphate pH 7.5, 500 mM NaCl, 10 μM ZnCl_2_, 10% glycerol, 2.5–250 mM imidazole, 0.1% Tween-20, 10 mM β-mercaptoethanol, 1% PMSF), glutathione (GSTrap HP; 50 mM Na-phosphate pH 7.5, 300 mM NaCl, 10 μM ZnCl_2_, 10% glycerol, 0.1% Tween-20, 2 mM DTT, 1% PMSF) and again Ni-NTA affinity chromatography. Highly pure fractions were dialysed against storage buffer (10 mM Tris-HCl pH8, 50 mM NaCl, 10% glycerol), snap frozen and stored at −80 °C.

Gel filtration was performed using a Superdex 200 10/300GL column (GE Healthcare) and an ÄKTA Purifier 10 (GE Healthcare) according to the manufacturer's instructions. Ni-NTA-enriched fractions were prepared as described above. Ni-NTA elution fractions were pooled, concentrated to 8 mg ml^−1^ using Amicon Ultra Centrifugal Filters (Millipore) and buffer was changed to gel filtration running buffer (50 mM Na-phosphate pH 7.5, 500 mM NaCl, 20% glycerol). Four milligrams of the enriched fraction was then loaded onto the gel filtration column. Column washing, loading and sampling of the fractions was done according to the manufacturer's instructions. Fractions (0.5 ml) were collected and 20 μl of each fraction was used for SDS–PAGE and western blot analysis.

To study the interaction of TDG and TET1, Ni-NTA and GST pull-down assays were performed. TDG–GST was co-expressed with a TET1 N-terminal fragment (His6–TET1-N aa 301–1366) or the TET1 catalytic domain (His6–TET1_CD_ aa 1367–2057) in *E. coli* as described above. Five milligrams of cleared *E. coli* lysate was then incubated with 25 μl of Glutathione Magnetic Beads (Thermo Scientific) or Ni-NTA Sepharose beads (Roche) in binding buffer (50 mM Na-phosphate pH 7.5, 300 mM NaCl, 20% glycerol, 0.1% Tween-20, 1 mM DTT, 1 mM PMSF) in a total volume of 1 ml at room temperature for 2 h. The beads were rinsed three times with 500 μl binding buffer and bound proteins were analysed by SDS–PAGE and western blotting.

Partial purification of TET1_CD_–TDG for activity assays was done via Ni-NTA affinity purification as described above. As catalytic mutants, His6–TET1_CD_Δcat (H1652Y; D1654A) and TDGΔcat–GST (N151A) were used.

### Analytical gel electrophoresis and western blotting

Protein fractions were analysed by standard SDS–PAGE followed by Coomassie blue staining or by immunoblotting using chemiluminescence (WesternBright ECL, Advansta) according to the manufacturer's protocol. Antibodies were diluted in 5% non-fat dry milk TBS (100 mM Tris-HCl pH 8 and 150 mM NaCl) supplemented with 0.2% Tween-20.

### Yeast two-hybrid assay

To confirm the interaction between TET1 and TDG, yeast two-hybrid assay was performed using he Matchmaker yeast-two hybrid system (Clontech). TET1 was divided into four overlapping fragments (TET1-1 aa 1–491; TET1-2 aa 397–931; TET1-3 aa 870–1403; TET1-4 aa 1367–2057) that were cloned into the BD (pAS2.1 BD FLAG) of the Gal4 protein and TDG was cloned into the AD (pACT2 AD) of the Gal4 protein. The *Saccharomyces cerevisiae* strain AH109 was co-transformed with 50–500 ng of bait and prey plasmids according to the Clontech manual. Interactions were assessed by spotting serial dilutions of cells on selective medium (SC-LEU-TRP-ADE-HIS) supplemented with 2.5 mM 3AT (3-Amino-1,2,4-triazole), a competitive inhibitor of the HIS3 gene product. Cells were incubated at 30 °C for 6–7 days.

### Base release assay

The catalytic activity of TET1–TDG was monitored by means of a standardized nicking assay^31^. Briefly, the reactions were carried out in a reaction volume of either 40 μl when using partially purified TET1–TDG from Ni-NTA affinity purification fractions or 20 μl when using purified recombinant protein containing TET reaction buffer (50 mM HEPES pH 8, 50 mM NaCl, 1 mM disodium-ketoglutarate, 2 mM ascorbic acid, 75 μM Fe(II) and 1 mM ATP), 0.5 pmol of substrate and 10 μl of Ni-NTA pulldown or 2 pmol purified TET1_CD_ and 1 pmol purified TDG (preincubated together on ice for 5 min), respectively. Reactions were incubated at 37 °C for 1 h and stopped by addition of 1 M NaOH to a final concentration of 100 mM and heating for 10 min at 99 °C. After ethanol precipitation at −20 °C overnight, the products were separated in a 15% denaturing polyacrylamide gel and labelled DNA was detected using the red or blue fluorescence mode of the Typhoon 9400 (GE Healthcare) and analysed quantitatively by ImageQuant TL software (v7.0, GE Healthcare).

TDG time-course reactions were carried out in 200 μl reaction volume containing nicking buffer (50 mM Tris-HCl pH 8, 1 mM DTT, 0.1 mg ml^−1^ BSA and 1 mM EDTA), 5 pmol of labelled substrate DNA and 5 pmol of purified TDG. After the indicated times of incubation at 37 °C, 20 μl aliquots were withdrawn and the reactions were stopped by the addition of 1 M NaOH to an end concentration of 100 mM and heating for 10 min at 99 °C. Reaction products were analysed by denaturing PAGE and analysed as described above.

### *In vitro* methylation and oxidation of plasmid DNA and slot blot analysis

*In vitro* methylation of pUC19 plasmid DNA was performed using M.SssI CpG methyltransferase (New England Biolabs) according to the manufacturer's instructions.

For the *in vitro* oxidation, 200 ng of methylated plasmid was incubated with 500 ng purified His6–TET1_CD_ from *E. coli* (see above). The reaction was carried out in TET reaction buffer (50 mM HEPES pH 8, 50 mM NaCl, 1 mM disodium-ketoglutarate, 2 mM ascorbic acid, 75 μM Fe(II) and 1 mM ATP) and incubated at 37 °C for 1 h. Reaction was stopped with the addition of NaOH and EDTA to a final concentration of 400 and 10 mM, respectively, and heating at 99 °C for 10 min. The denatured DNA was blotted using the Bio-Rad slot blot system according to the manufacturer's instruction. Hybond-N+ nylon membranes (Amersham) were ultraviolet cross-linked, blocked with 5% milk and immunostaining against 5mC, 5hmC, 5fC and 5caC, and was performed using chemiluminescence (WesternBright ECL, Advansta) according to the manufacturer's protocol. Antibodies were diluted in 5% non-fat dry milk TBS (100 mM Tris-HCl pH 8 and 150 mM NaCl) supplemented with 0.2% Tween-20.

### LC–MS/MS analysis

Plasmid DNA samples were enzymatically hydrolysed to deoxyribonucleosides in a two step reaction. First DNA was incubated at 45 °C for 40 min in 10 mM ammonium acetate buffer pH 5.3 containing 5 mM magnesium chloride and 0.2 U nuclease P1 from Penicillium citrinum (Sigma, N8630). The samples were then buffered in ammonium bicarbonate to a final concentration of 100 mM and incubated at 37 °C for 30 min with 0.0002 U phosphodiesterase I from *Crotalus adamanteus* venom (Sigma, P3243) and 0.3 U alkaline phosphatase from *E. coli* (Sigma, P5931). The reactions were stopped and contaminants, which could potentially clog the HPLC column, were precipitated by adding three volume equivalents of ice-cold acetonitrile and centrifugation at 16,000 *g* for 30 min. The supernatants were collected in new tubes and vacuum centrifuged at room temperature until dry. Salt residues, originating from buffers, were partially evaporated by re-dissolving the samples in 100 μl of water and vacuum drying one more time. The standards for 5-me(dC), 5-hm(dC), 5-ca(dC) and 5-f(dC) were prepared to contain the same amount of salts as the samples and followed the same desalting procedure. The samples were then finally dissolved in 50 μl of water for LC–MS/MS analysis of 5-me(dC), 5-hm(dC), 5-ca(dC) and 5-f(dC). For quantification of unmodified nucleosides (dA, dC, dG and dT), samples were diluted 1:10 with water. For some of the samples, 1:10 dilution was also used during quantification of 5-me(dC). Quantification was performed with the use of an LC-20AD HPLC system (Shimadzu) coupled to an API 5000 triple quadrupole (ABSciex) operating in positive electrospray ionization mode. The chromatographic separation was performed at 40 °C with the use of an Ascentis Express C18 2.7-μm 150 × 2.1 mm i.d. column protected with an Ascentis Express Cartridge Guard Column (Supelco Analytical) with an Exp Titanium Hybrid Ferrule (Optimize Technologies Inc.). The mobile phase consisted of A (water and 0.1% formic acid) and B (methanol and 0.1% formic acid) solutions. The following conditions were employed during chromatography: for unmodified nucleosides, 0.13 ml min^−1^ flow, starting at 10% B for 0.1 min, ramping to 60% B over 2.4 min and re-equilibrating with 10% B for 4.5 min; for 5-me(dC), 5-hm(dC), 5-ca(dC) and 5-f(dC), 0.14 m l min^−1^ flow, starting at 5% B for 0.5 min, ramping to 45% B over 8 min and re-equilibrating with 5% B for 5 min. For mass spectrometry detection, the multiple reaction monitoring was implemented using the following mass transitions: 252.2/136.1 (dA), 228.2/112.1 (dC), 268.2/152.1 (dG), 243.2/127.0 (dT), 242.1/126.0 [5-me(dC)], 258.1/142.0 [5-hm(dC)], 256.1/140.0 [5-f(dC)] and 272.1/156.0 [5-ca(dC)].

### DSB assay

DSB assays were carried out in 20 μl reaction volumes containing incision buffer (50 mM HEPES pH 8, 70 mM KCl, 7 mM MgCl_2_, 500 μg ml^−1^ BSA and 1 mM DTT), 0.5 pmol of labelled substrate, 1 pmol APE1 and 1 pmol TDG. After incubation at 37 °C for 30 min, proteinase K was added to a final concentration of 100 μg ml^−1^ and the reaction was incubated at 37 °C for 30 min. Samples were then separated on 8% native polyacrylamide gels, and detected and quantified.

### BER reconstitution

The BER reconstitution reaction was carried out stepwise to analyse individual stages of the process. The reaction mixture containing 1 pmol labelled 60 or 59 bp DNA, 5 pmol His6-TET1_CD_ and 2 pmol His6–TDG were incubated at 37 °C for 30 min in TET reaction buffer (50 mM HEPES pH 8, 50 mM NaCl, 1 mM disodium-ketoglutarate, 2 mM ascorbic acid, 75 μM Fe(II) and 1 mM ATP), to generate an AP-site. The reaction mixture was then supplemented with 70 mM KCl, 7 mM MgCl_2_, 200 μM dCTP or dNTP, 2 mM ATP, 500 μg ml^−1^ BSA, 1 mM DTT and 10 pmol APE1 and incubated at 37 °C for 5 min. DNA polβ (0.5 pmol) was then added and the reaction mixture incubated for a further 5 min. Finally, 2 pmol XRCC1–LIG3 complex was added for a 10-min incubation. Reactions were terminated by the addition of stop buffer (50 mM Tris-Cl pH 8, 0.5% SDS and 100 mM NaBH_4_) and incubation on ice for 20 min. The reaction products were analysed by denaturing PAGE and analysed as described above.

For the analysis of the endproduct with HpaII or MscI endonuclease digest, the reconstitution reaction was carried out by adding all the factors at the same time and incubation at 37 °C for 1 h followed by ethanol precipitation of the labelled DNA at −20 °C overnight. The recovered DNA was then treated with a total of 5 U HpaII or MscI endonuclease (New England Biolabs) at 37 °C for 60 min, fragments were separated in 8% native polyacrylamide gels and detected as described above.

## Additional information

**How to cite this article:** Weber, A. R. *et al*. Biochemical reconstitution of TET1–TDG–BER-dependent active DNA demethylation reveals a highly coordinated mechanism. *Nat. Commun.* 7:10806 doi: 10.1038/ncomms10806 (2016).

## Supplementary Material

Supplementary Information Supplementary Figures 1-5
