# Supplementary material for: Biochemical reconstitution of TET1–TDG–BER-dependent active DNA demethylation reveals a highly coordinated mechanism
Source: Nat Commun. 2016 Mar 2;7:10806. doi: 10.1038/ncomms10806 (PMC4778062; doi:10.1038/ncomms10806)
Supplement: Supplementary Information — Supplementary Figures 1-5 [file ncomms10806-s1.pdf]

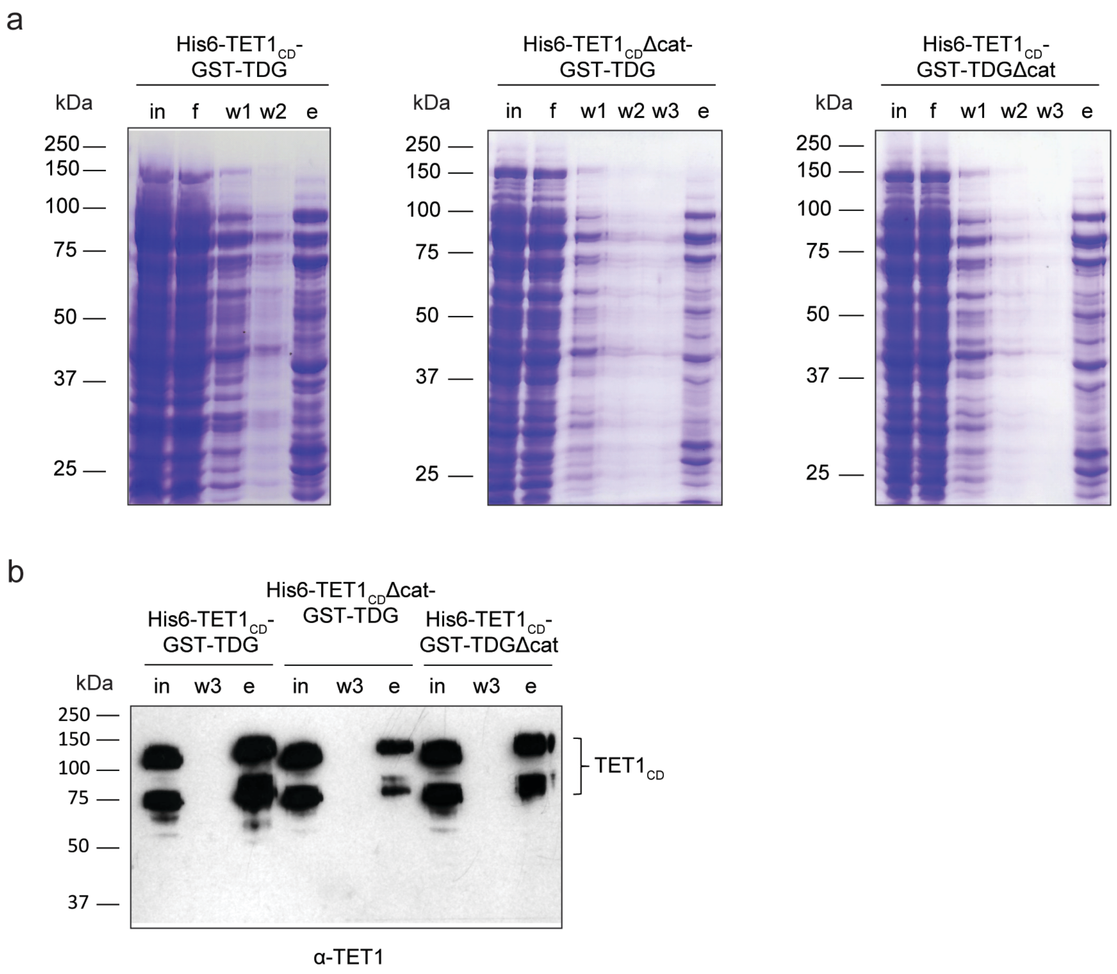

Supplementary Figure 1: Ni-NTA affinity purification of *E.coli* lysates co-expressing His6-TET1$_{CD}$ and TDG-GST. After co-expression of His6-TET1$_{CD}$ and TDG-GST (250 µM IPTG, 25°C for 3 h) cells were lysed and Ni-NTA affinity purification was carried out using Ni-NTA Sepharose beads according to the manufacturer's instructions. Fractions were analyzed by SDS-PAGE followed by (**A**) Coomassie staining or (**B**) immunoblotting using anti TET1 antibody (Millipore). An immunoblot of TDG from Ni-NTA co-affinity purification can be found in Fig. 1. in = input, f = flow, w = wash, e = elution.

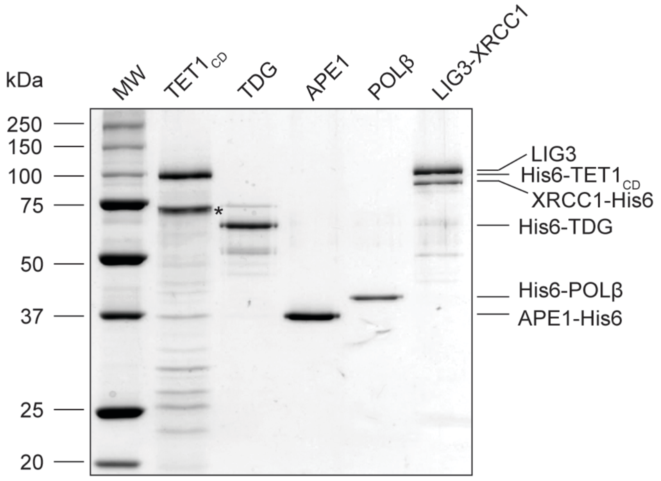

Supplementary Figure 2: Purified His6-TET1$_{CD}$ and BER proteins used in biochemical assays. Purified His6-TET1$_{CD}$, His6-TDG, APE1-His6, His6-POLβ, LIG3-XRCC1-His6 used in the biochemical assays were analyzed by SDS-PAGE followed by Coomassie staining. Purification procedures are described in Materials and Methods. Except for LIG3 the affinity tags were not cleaved off. * catalytically inactive C-terminal TET1$_{CD}$ fragment.

a

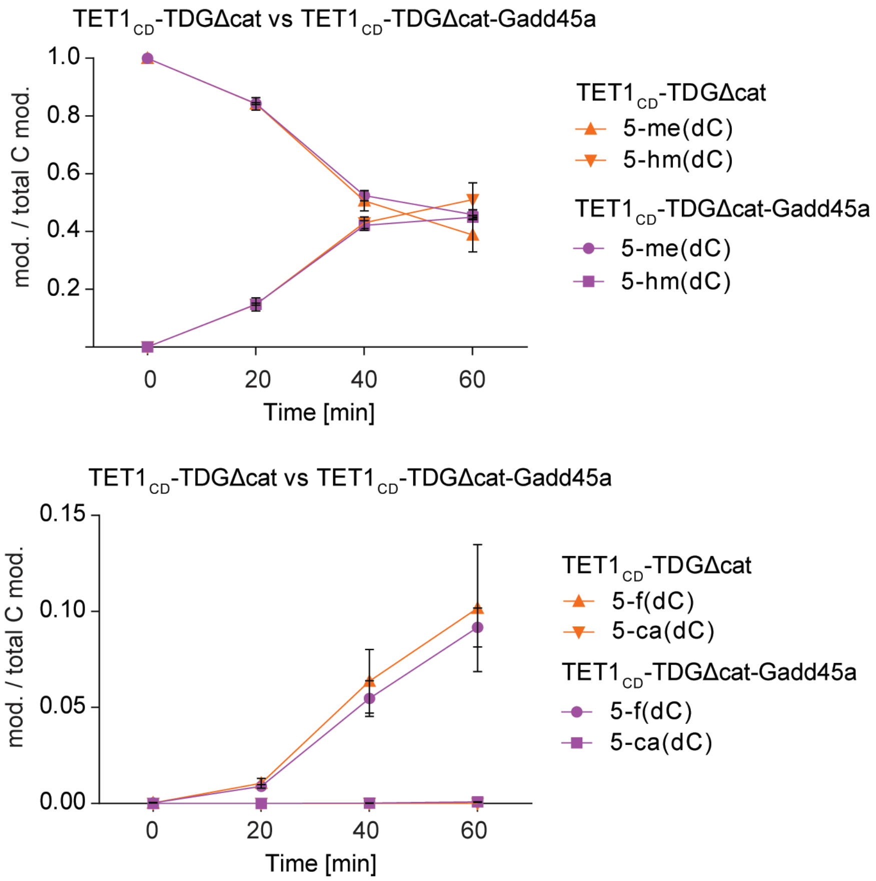

b

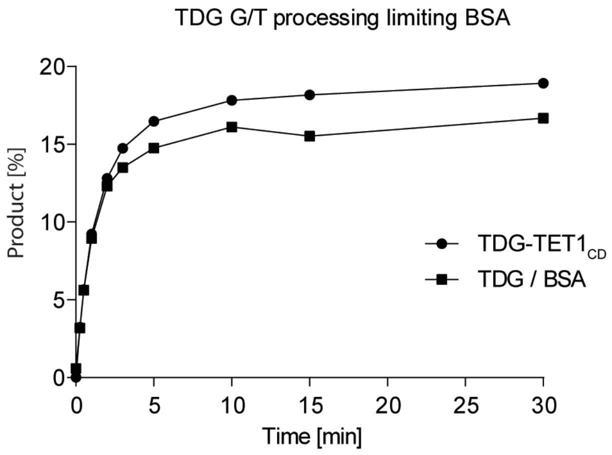

Supplementary Figure 3: (**a**) Gadd45a does not stimulate TET1$_{CD}$ in complex with TDGΔcat. *In vitro* methylated pUC19 plasmid was treated with a His6-TET1$_{CD}$-His6-TDGΔcat or His6-TET1$_{CD}$-His6-TDGΔcat-His6-Gadd45a and resulting cytosine modifications were analyzed by LC/MS/MS (n = 3, normalized mean values with standard deviations). (**b**) The effect of His6-TET1$_{CD}$ on His6-TDG catalysis assessed in a base release assay under multiple turnover conditions. A 5-fold molar excess of G•T substrate (125 nM) over TDG protein (25 nM) (lower panel) was incubated at 37°C and

reactions stopped by the addition of NaOH after the indicated times. Product formation was monitored and quantified using denaturing gel electrophoresis and fluorescent scanning.

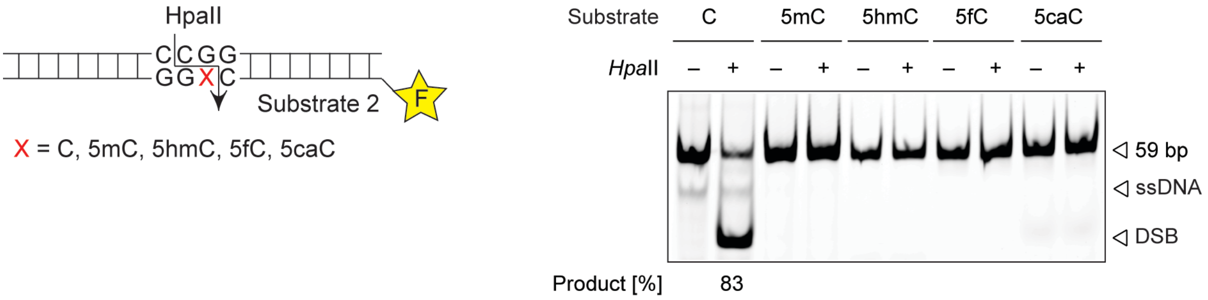

Supplementary Figure 4: *Hpa*II cleavage requires fully unmodified CCGG sequences. Endonuclease digest using *Hpa*II (10 U) on a labeled 59 bp substrate containing either a single 5mC, 5hmC, 5fC, 5caC or an unmodified C at the CpG of the *Hpa*II recognition site (CCGG). Reaction products were analyzed by native polyacrylamide gel electrophoresis to monitor the appearance of DSBs. The positions of the 59 bp substrate DNA and product fragment are indicated; ssDNA, free single-stranded DNA.

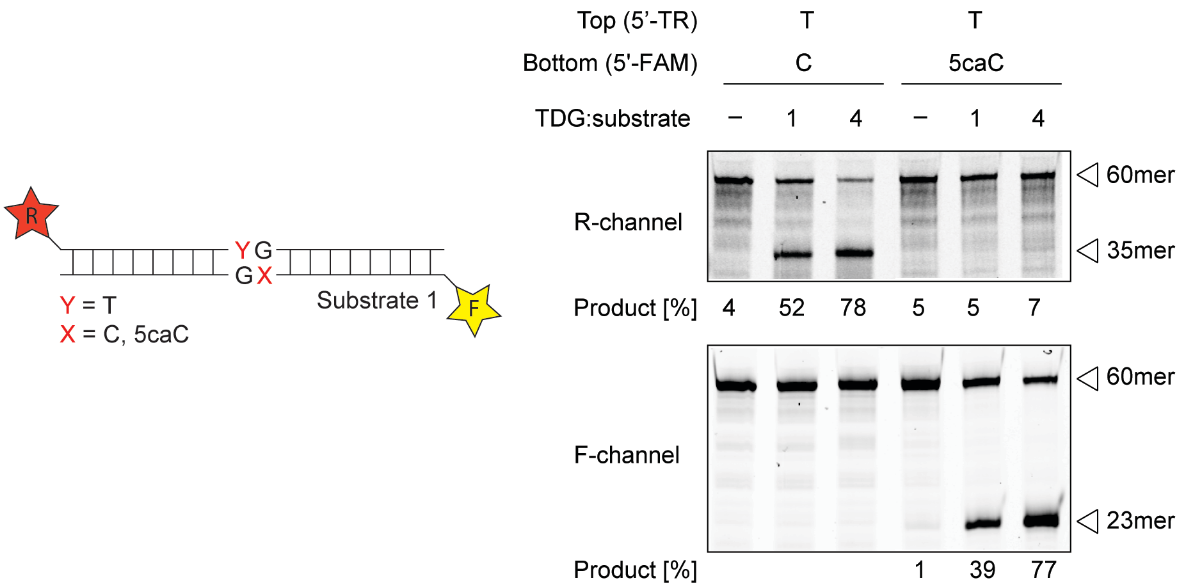

Supplementary Figure 5: Processing of differentially modified CpGs by TDG. Base release assay with His6-TDG on labeled 60 bp substrates containing T on the labeled top strand (5’ TexasRed, TR) and 5caC or C on the labeled bottom strand (5’ fluorescein, FAM) within the CpG context, illustrated at the top. Reactions were carried out at 37°C for 15 min with the indicated enzyme/substrate ratio, stopped by the addition of NaOH and separated by denaturing gel electrophoresis. Both strands were then visualized by fluorescent scanning and quantified.
